# Empyroform, a New Tar Preparation

**Published:** 1905-09

**Authors:** 


### Empyroform, a New Tar Preparation.

Dr. F. Kornfeld, assistant at Prof. von Fritsch's polyclinic, Vienna. (*Centralblatt f.d. gesamte Therapie,* No. 12, 1904.) Incited by the favorable results reported by the clinics of Neisser, Pick, and von Duering, I tested empyroform, which is a condensation product of birch tar and formalin, in my own dermatological practice. The most satisfactory results were gotton from a 5 to 20 per cent. empyroform-vaselin ointment, a 5 to 20 per cent. empyroform-Lassar's paste, a 50 per cent. empyroform-vaselin paste, and a 5 to 15 per cent. liniment. Of much value was also the dry paint of empyroform ½ oz.; talc. venet. 2½ drams; glycerin 2½ drams; aq. dest. 5 drams, well shaken before use.

Even the 50 per cent. paste smells but slightly of tar, and only very little shading of the skin occurs. In contradistinction to tar, it causes neither local irritation nor systemic intoxication.

We used it in a whole series of the most varied chronic eczemas, with good results, and never had a relapse. Thus encouraged, we applied it in cases of less torpid, subacute inflammation; and the course of these was also influenced most favorably. Some of them had been absolutely intolerant of tar in the past; but we found that they could use it without reaction after a course of empyroform. Empyroform first caused retrogression of the redness and infiltration; the itching, burning and tension became less; and in an astonishingly short time there was complete cure. This was especially seen in those obstinate cases that had proved recalcitrant to the usual measures, including tar.

It proved effectual in a large number of weeping eczemas of the hands, fingers, fore-arms and face, even when fresh. In seborrheal eczema surprisingly good effects were noted, and these must be attributed to the fact that it contains the disinfectant formalin in addition to the reducing agent tar. Much benefit was also experienced in prurigo, psoriasis and lichen urticatus.

